# Tick bite presenting with acute abdomen

**DOI:** 10.11604/pamj.2016.24.70.9463

**Published:** 2016-05-20

**Authors:** Rakesh Sharma, Susanta Meher

**Affiliations:** 1 Department of Surgery AIIMS, Bhubaneswar, Odisha 751019, India

**Keywords:** Tick bite, acute abdomen, emergency

## Image in medicine

A 24year old man presented to the emergency department with complain acute onset pain abdomen for 3days. The pain started in the umbilical region, later became diffuse. There was no history of fever, vomiting or irregular bladder and bowel habits. He consulted twice in the nearby hospital for pain abdomen. Routine blood investigation, including ultrasonography of the abdomen and pelvis was also done which did not reveal any significant abnormality. He was given some oral medication to relieve his pain, but he didn't get relieved of the pain. He then presented to our emergency department with intense and diffuse abdominal pain. Initial evaluation revealed features of diffuse peritonitis. Careful examination of the abdomen revealed a tick biting at the umbilicus (A). The tick was tense with full of blood and appeared dark bluish in color. The tick was removed and the patient got relieved of symptoms within next few minutes (B). Tick are spider like animals that bites to fasten themselves to the skin to feed on blood to grow and survive. Tick bite normally don't cause any symptoms. During bite, they secrete a neurotoxin which prevents the host from feeling the pain. Sometimes intense pain can occur before or after the tick drops off. Minor flu like symptoms can occur during tick bite like fever, headache, nausea, vomiting and malaise including features of local irritation. Rarely tick bite can cause severe allergic reaction and muscle paralysis.

**Figure 1:**
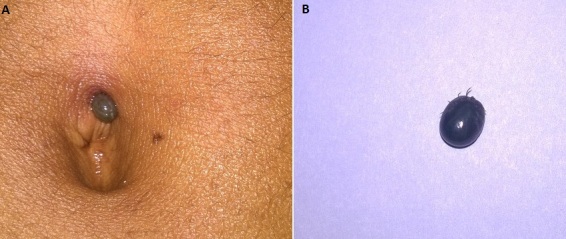
A) tick bitting at the umbilicus; B) engorged tick after removal from the umbilicus

